# Editorial: The complex phenotype of diabetic cardiomyopathy: clinical indicators and novel treatment targets, volume II

**DOI:** 10.3389/fendo.2025.1752170

**Published:** 2025-12-11

**Authors:** Priyanka Choudhury, Yin Cai

**Affiliations:** 1 Department of Pediatrics, Medical College of Wisconsin, Milwaukee, WI, United States; 2 Department of Health Technology and Informatics, The Hong Kong Polytechnic University, Hong Kong, Hong Kong SAR, China

**Keywords:** diabetic cardiomyopathy, multimodality imaging, biomarker, lipid metabolism, angiogenesis

Diabetic cardiomyopathy (DCM) is a complex cardiovascular complication of diabetes mellitus, characterized by metabolic, structural, and functional abnormalities of the myocardium that occur independently of coronary artery disease or hypertension 1, 2). As global rates of type 2 diabetes mellitus (T2DM) continue to rise, the incidence of DCM is expected to increase substantially, underscoring the urgent need for deeper mechanistic insights, earlier diagnostic approaches, and targeted therapeutic strategies. This second volume of our Research Topic brings together diverse studies that collectively advance our understanding of DCM pathogenesis and clinical management, integrating perspectives from innovative preclinical models, advanced imaging, molecular investigations, and precision risk stratification tools.

One of the major contributions in this volume is the development of a combined nicotinamide/streptozotocin-induced T2DM and carotid vascular balloon injury (VBI) model in Sprague–Dawley rats (Wang et al.). Through percutaneous transluminal coronary angioplasty balloon catheterization, this model successfully induces rapid hyperglycemia, marked neointimal proliferation, and diminished plaque stability, overcoming key limitations of traditional DCM models that rely on genetic manipulation or longer induction periods. Importantly, rats exposed to both T2DM and VBI displayed vascular pathology at two weeks comparable to six-week outcomes in VBI-only controls, providing a valuable accelerated disease system for evaluating therapeutic interventions (Wang et al.).

Complementing these preclinical developments, several clinical studies included in this volume reinforce the interaction between metabolic disturbances and cardiac pathology in diabetes. CT-derived visceral fat area (VFA) was independently associated with cardiovascular disease in T2DM patients, emphasizing the role of abdominal adiposity in cardiovascular risk (Wu et al.). Multiparametric cardiac magnetic resonance imaging (CMR) further demonstrated that impaired glucose metabolism is linked to increased myocardial fibrosis and subclinical inflammation in patients with hypertrophic cardiomyopathy (HCM) (Peng et al.). Prediabetic and diabetic cohorts showed progressively elevated myocardial T1 values and extracellular volume fractions (ECV), with strong positive correlations between ECV and HbA1c levels. Raised T2 values supported the presence of underlying inflammation. These effects were most pronounced in hypertrophied myocardial regions, highlighting the detrimental influence of even mild hyperglycemia on myocardial remodeling and supporting the early incorporation of metabolic control and CMR-based tissue characterization into HCM management (Peng et al.).

Sex-specific considerations also emerge in the analysis of insulin resistance and hypertension risk. A retrospective cohort study examining the triglyceride–glucose (TyG) index found that while the association between TyG and incident hypertension in men weakened after adjusting for confounders, spline analyses revealed stronger risk relationships in men than women (Sun et al.). Sensitivity analyses partially validated these findings, suggesting that biological sex may modulate hypertension risk associated with insulin resistance and pointing to a need for larger studies to guide personalized prevention strategies.

This volume also highlights the use of predictive modeling and bioinformatics to assess DCM risk. A nomogram developed by Luo et al., integrating age, duration of diabetes, systolic blood pressure, urinary albumin-to-creatinine ratio, left atrial diameter, and left ventricular posterior wall thickness, demonstrated excellent discrimination and calibration for predicting DCM. By leveraging routine clinical and echocardiographic parameters, the model provides an accessible tool for individualized risk stratification and early cardioprotective intervention.

At the molecular level, studies in this volume identify key regulators of angiogenesis and lipid metabolism as contributors to DCM pathogenesis. High-glucose stimulation significantly upregulated ephrinB2 (Efnb2), which sequencing analyses suggest may mediate angiogenic impairment in diabetic conditions (Hu et al.). Integrative bioinformatics identified two hub genes, Acsbg1 and Etnppl, out of intersecting lipid metabolism–related and DCM-associated gene sets (Huang et al.). Acyl-CoA Synthetase Bubblegum Family Member 1 (Acsbg1) was strongly upregulated in disease models, while ethanolamine-phosphate phospho-lyase (Etnppl) expression was reduced but recoverable with therapeutic intervention. Both genes localized to UCAGG motifs in RNA secondary structures and appeared to exert their effects through lysosomal pathways. These findings link dysregulated lipid metabolism to DCM progression and position Acsbg1 and Etnppl as promising diagnostic and therapeutic targets (Huang et al.).

Imaging and biomarker advancements further enhance precision in early DCM detection. Multimodality imaging techniques, including echocardiography, CMR, and nuclear imaging, continue to refine the evaluation of diastolic dysfunction, early hypertrophy, and myocardial fibrosis (Adel and Chen). Circulating biomarkers such as soluble suppression of tumorigenicity 2 (sST2), cardiotrophin-1 (CT-1), galectin-3, lysyl oxidase-like 2 (LOXL2), and electron transfer flavoprotein β (ETFβ) demonstrated strong associations with DCM severity and improved diagnostic accuracy when integrated with conventional indices such as ultrasound E/E′ ratio and NT-proBNP (Su et al.). Noninvasive techniques like skin autofluorescence also show emerging potential in risk assessment.

A unifying mechanistic perspective across several contributions is the central role of mitochondrial calcium dysregulation in DCM (Deng et al.). Impaired mitochondrial calcium uptake, excessive release, and reduced buffering capacity lead to bioenergetic collapse, oxidative stress, and cardiomyocyte injury. By linking metabolic stress and mitochondrial dysfunction, this framework highlights a key mechanistic hub in DCM development. Deng et al. further evaluate therapeutic strategies targeting the mitochondrial calcium uniporter (MCU), mitochondrial Na^+^/Ca²^+^/Li^+^ exchanger (NCLX), and mitochondrial permeability transition pore (mPTP), offering critical insights into their translational viability.

Collectively, the studies in this volume highlight diabetic cardiomyopathy as a complex disorder shaped by interacting metabolic, vascular, inflammatory, and mitochondrial disturbances. By integrating accelerated animal models, advanced imaging, molecular analyses, and predictive tools, these contributions deepen our mechanistic understanding and strengthen the translational framework for improving early diagnosis and treatment.

Looking ahead, advancing diabetic cardiomyopathy research will require refined, physiologically relevant disease models to probe early pathogenesis and test therapies, alongside longitudinally validated multimodality imaging and circulating biomarkers for subclinical detection. Molecular targets, including regulators of lipid metabolism, angiogenesis, and mitochondrial calcium handling, merit further investigation, while predictive algorithms based on clinical and imaging measures should be expanded to support personalized risk assessment.

In parallel, nanoparticle-based therapeutics offer promising opportunities for organelle-specific drug delivery, enhanced stability, and modulation of key pathways in inflammation, angiogenesis, mitochondrial function, and fibrosis (3, 4). Future work should define molecular mechanisms of regeneration, mitochondrial homeostasis, immune activation, and extracellular matrix remodeling in diabetes, while rigorously evaluating nanoparticle biodistribution, immune responses, clearance, and toxicity. Integrating nanotechnology with mechanistic insights from metabolism, vascular biology, and mitochondrial signaling may enable precision therapies that prevent or reverse remodeling, ultimately shifting the trajectory of DCM rather than merely mitigating its consequences.
